# MicroRNA-30d and microRNA-181a regulate HOXA11 expression in the uterosacral ligaments and are overexpressed in pelvic organ prolapse

**DOI:** 10.1111/jcmm.12448

**Published:** 2015-01-29

**Authors:** Myung Jae Jeon, Eun Jae Kim, Maria Lee, Hoguen Kim, Jong Rak Choi, Hee Dong Chae, Yeo Jung Moon, Sei Kwang Kim, Sang Wook Bai

**Affiliations:** a Department of Obstetrics and Gynecology, Seoul National University College of MedicineSeoul, Korea; b Department of Pathology, Yonsei University College of MedicineSeoul, Korea; c Department of Laboratory Medicine, Yonsei University College of MedicineSeoul, Korea; d Department of Obstetrics and Gynecology, Asan Medical Center, University of Ulsan College of MedicineSeoul, Korea; e Department of Obstetrics and Gynecology, Yonsei University College of MedicineSeoul, Korea

**Keywords:** HOXA11, microRNA, pelvic organ prolapse, uterosacral ligament

## Abstract

The balanced turnover of collagen is necessary to maintain the mechanical strength of pelvic supportive connective tissues. Homeobox (HOX) A11 is a key transcriptional factor that controls collagen metabolism and homoeostasis in the uterosacral ligaments (USLs), and the deficient HOXA11 signalling may contribute to alterations in the biochemical strength of the USLs, leading to pelvic organ prolapse (POP). However, it is unknown how HOXA11 transcripts are regulated in the USLs. In this study, we found that microRNA (miRNA)-30d and 181a were overexpressed in women with POP, and their expression was inversely correlated with HOXA11 mRNA levels. The overexpression of miR-30d or 181a suppressed HOXA11 mRNA and protein levels in 293T cells, whereas the knockdown of these miRNAs enhanced HOXA11 levels and collagen production. Cotransfection of a luciferase reporter plasmid containing the 3′-untranslated region of HOXA11 with miR-30d or 181a mimic resulted in decreased relative luciferase activity. Conversely, cotransfection with anti-miR-30d or 181a increased luciferase activity. Taken together, these results indicate that both miR-30d and 181a are important posttranscriptional regulators of HOXA11 in the USLs and could be a potential therapeutic target for POP.

## Introduction

Pelvic organ prolapse (POP) is the downward descent of pelvic organs that results in a protrusion of the vagina, uterus or both. While not life-threatening, it often causes bladder, bowel and pelvic symptoms that can have an adverse effect on a woman's daily activities and quality of life 1. POP is also a significant economic burden to women and healthcare systems. It affects almost half of all women over 50 years of age 2, and one in five women will undergo surgery for POP in their lifetime 3. In the United States, the direct cost of prolapse surgery is greater than 1 billion dollars per year 4, and as the population is ageing, the demand for POP care is estimated to double over the next 40 years 5.

Nonetheless, relatively little is known about the underlying pathophysiology of POP. Although several factors, including advancing age, vaginal childbirth and obesity, have been implicated as the risk factors, they do not fully explain the development of POP. It has been proposed that the biomechanical weakness of pelvic floor supportive structures, compounded by the risk factors cited above, leads to POP 1. Moreover, the need to improve treatment strategies is highlighted by the high failure rate after surgery, and this is related to the poor quality of pelvic supportive tissues 6. Therefore, the identification of critical molecular pathways involved in the attenuation of pelvic supportive tissues is essential for developing effective prevention and treatment strategies for POP.

Recently, Homeobox (HOX) A11 has attracted great attention for its role in the development and maintenance of the uterosacral ligaments (USLs), the main supportive structure of the uterus and upper vagina. Targeted deletion of this gene in HOXA11 null-mutant mice resulted in the lack of the USLs 7. In another murine model, repression of HOXA11 by vectors bearing HOXA11 short hairpin RNA led to the attenuation of the USLs, concomitant with changes in collagen metabolism favouring catabolism over synthesis 8. HOXA11 has also been reported to promote the proliferation of fibroblasts, the major cell type that produces collagen, *in vitro* 9. Thus, HOXA11 is now characterized as a key upstream transcriptional factor that controls collagen metabolism and homoeostasis in the USLs, and the deficient HOXA11 signalling may contribute to alterations in the biochemical strength of the USLs, leading to POP 7–9. However, it is unknown how HOXA11 transcripts are regulated in the USLs.

MicroRNAs (miRNAs) are small (∽22 nucleotides), endogenous RNA molecules that play important regulatory roles by targeting mRNAs for cleavage or translational repression. Although miRNA genes represent nearly 1% of the genome, it is estimated that ∽30% of the protein-encoding genes are regulated by at least one miRNA 10,11. They play key roles in diverse regulatory pathways including developmental processes, cell growth, differentiation and apoptosis 12, and it has been reported that aberrant miRNA expression is associated with various human diseases 13,14. However, the role of miRNAs in the pathogenesis of POP has not been addressed. Considering the emerging evidence showing that miRNAs regulate several other HOX genes 15, aberrantly expressed miRNAs may contribute to the development of POP by modulating the expression of HOXA11. To test this hypothesis, we selected miRNAs based on microarray and a bioinformatic approach 16, and then verified their expressions and the correlation with HOXA11 expression in the tissues using further quantitative real-time PCR (qRT-PCR) analysis. We also performed a series of functional studies to determine the role of those miRNAs in the regulation of HOXA11.

## Materials and methods

### Tissue collection

All experiments were performed following the approval of the review board for human research of Seoul National University Hospital (H-1302-087-466), and informed consent was obtained from all of the participating women. Prior to surgery, a pelvic examination using the POP-quantification system was performed to evaluate the presence of POP. Data regarding age, vaginal parity, body mass index, menopausal status and prior prolapse surgery were also recorded. Menopause was defined as the cessation of menses for at least 1 year.

The patient group consisted of 38 women who underwent hysterectomy with sacrocolpopexy for symptomatic POP that was stage II or greater. The control group consisted of 38 age-matched women with POP-quantification stage 0 or I who received operations for benign gynaecological indications (*i.e*. leiomyoma, adenomyosis, endometrial hyperplasia, carcinoma *in situ* of the uterine cervix). There were no differences in mean age, vaginal parity, body mass index or menopausal status between the two groups (Table1). None of the postmenopausal women in either group were undergoing hormone replacement therapy. At the time of surgery, USL samples 1 × 1 cm in size were collected from the area of insertion into the cervix, a location where the ligament is consistently identifiable. The samples were immediately snap frozen in liquid nitrogen and kept at −80°C until RNA extraction was performed. From each group, eight samples were used for microarray, and the remaining 30 samples were used for qRT-PCR validation.

**Table 1:** Clinical characteristics of enrolled women with and without POP

	Patient (*n* = 38)	Control (*n* = 38)	*P*-value
Age (year), mean ± SD	56.0 ± 9.5	54.1 ± 7.9	0.35
Vaginal parity, median (IQR)	2 (1)	2 (1)	0.21
BMI (kg/m^2^), mean ± SD	24.8 ± 3.0	24.4 ± 3.3	0.66
Menopause, *n* (%)	26 (68.4)	21 (55.3)	0.24
Prior prolapse surgery, *n* (%)	0	0	NA
POP-Q stage, *n* (%)			
0–I	0	38 (100)	<0.01
II	10 (26.3)	0	
III	20 (52.6)	0	
IV	8 (21.1)	0	

POP, pelvic organ prolapse; SD, standard deviation; IQR, interquartile range; BMI, body mass index; NA, not applicable; POP-Q, pelvic organ prolapse-quantification.

### MiRNA microarray

MiRNAs were extracted using the mirVana miRNA isolation kit (Ambion, Austin, TX, USA) according to the manufacturer's protocols. Purified miRNAs were labelled using the mirVana miRNA Array Labeling kit and coupled to the Cy5 Post-Labeling Reactive Dye (Amersham, GE Healthcare Bio-Sciences, Piscataway, NJ, USA). The labelled samples were washed and hybridized in duplicate to mirVana miRNA Bioarrays (Ambion) using the mirVana miRNA Bioarray Essentials kit. Fluorescence intensities were processed and measured using the GeneChip scanner 3000 7G (Agilent Technologies, Santa Clara, CA, USA). The levels of miRNA hybridization were determined using GenePix Pro 6.0 software as recommended by the manufacturer. The background-adjusted intensity for each miRNA was subjected to a global variance stabilization normalization procedure. MiRNAs were considered to be overexpressed only if the differences were determined to be significant by a two-sample *t*-test (*P* < 0.05) and on average showed at least a 1.5-fold increase in patient samples compared with matched controls. Heatmap analysis and hierarchical clustering were performed with R project software.

### Cell culture

293T cells, derived from human embryonic kidney, were purchased from the American Type Culture Collection (Manassas, VA, USA) and maintained in DMEM (Sigma-Aldrich, St Louis, MO, USA), containing 10% foetal bovine serum without antibiotics-antimycotics, at 37°C in a humidified 5% CO_2_ incubator until the cells reached 40–50% confluence. Culture media were replaced with fresh media every 2–3 days. The cells were used between passages 5 and 10.

### Transfection experiments

293T cells were plated at a cellular density of 1 × 10^6^ per well on 100-mm culture dishes and cultured overnight. The cells were then transfected with double-stranded RNA oligos comprising the mature miRNA (miRNA mimic), RNA oligonucleotides complementary to mature miRNA (anti-miRNA) or negative controls (miRNA mimic, anti-miRNA negative control; GenePharma Co., Ltd, Shanghai, China) at a final concentration of 100 nM with the use of Lipofectamine RNAiMAX reagent (Invitrogen, Carlsbad, CA, USA). At 48 hrs after transfection, cellular lysates were collected for RNA or protein isolation. The transfection efficiency was ∽80%, as measured by the uptake of a FAM-labelled negative control.

### qRT-PCR analysis

Total RNA was extracted using the mirVana miRNA isolation kit (Ambion), and then cDNA was generated using the GoScript RT system (Promega, Madison, WI, USA) according to the manufacturer's protocol. qRT-PCR for indicated genes was performed in a reaction mixture containing SYBR Premix Ex Taq (TaKaRa, Tokyo, Japan). PCR primer sequences were HOXA11, forward 5′-GTACTTACTACGTCTCGGGTCCAG-3′ and reverse 5′-AGTCTCTGTGCACGAGCTCCT-3′; and β-actin, forward 5′-CGTACCACTGGCATCTGAT-3′ and reverse 5′-GTGTTGGCGTACAGGTCTTTG-3′. The reaction mixtures were preincubated at 95°C for 5 sec. followed by 40 cycles of denaturation at 95°C for 5 sec., annealing at 60°C for 34 sec. and extension at 95°C for 15 sec., 60°C for 1 min. and 95°C for 15 sec. The quantification of miRNAs was carried out using TaqMan miRNA assays (Applied Biosystems, Carlsbad, CA, USA) according to the manufacturer's protocol. The samples were analysed with the 7500 real-time PCR system (Applied Biosystems). All PCRs were performed in triplicate, and the specificity of each reaction was determined by melting curve analysis at the dissociation stage. For relative quantification, the data were analysed using the 2^−ΔΔCt^ method, where β-actin and U6B were used as internal controls for HOXA11 and miRNAs, respectively.

### Western blot analysis

Cells were lysed in 200 μl of RIPA buffer [150 mM NaCl, 1% NP-40, 0.5% sodium deoxycholate, 0.1% sodium dodecyl sulphate, 50 mM Tris-HCl (pH 8.0), 100 mM PMSF] with a protease inhibitor and centrifuged at 14,000 × g for 10 min. at 4°C. The proteins in the supernatant were mixed with denaturing sample buffer (1:1) and boiled for 5 min. at 94°C. Equal amounts of protein (30 μg) were loaded and separated by 10% SDS-PAGE and blotted onto nitrocellulose membranes (Bio-Rad, Hercules, CA, USA). The membranes were blocked with TBST containing 5% nonfat dry milk for 1 hr at 4°C and incubated with anti-HOXA11 (1:1,000; Abcam, Cambridge, UK), COL1A2 (1:500; Abcam), or COL3A1 (1:500; Sigma-Aldrich) antibodies overnight at 4°C. An anti-β-actin antibody (1:5,000; Sigma-Aldrich) was used as a control. After washing in TBST, the membranes were incubated with horseradish peroxidase-conjugated secondary antibodies (Abcam) for 1 hr at 4°C and washed again in TBST. The signal was detected using an enhanced chemiluminescence kit (Thermo Scientific, Rockford, IL, USA) and the intensity was quantified using ImageJ software.

### Luciferase reporter assay

To validate the HOXA11 3′-untranslated region (UTR) as a target of miR-30d and 181a, *in vitro* assays using the miTarget miRNA 3′-UTR target clones (HmiT008983-MT01; Genecopoeia, Rockville, MD, USA) were performed. These miRNA target clones consisted of the pEZX-MT01 vector containing the coding sequences of both firefly and Renilla luciferase and the full 3′-UTR of the HOXA11 transcript (accession number: NM_005523.5) inserted downstream of the firefly luciferase sequence. According to TargetScan (www.targetscan.org), the binding sites of miR-30d and 181a are predicted to be located at positions 1173 to 1180 and 1219 to 1226, respectively. For mutagenesis assays, these two miRNA-binding sites within the 3′-UTR of the HOXA11 transcript were deleted. After heat-shock transformation in competent *Escherichia coli* cells (One Shot TOP 10 competent cells; Invitrogen), the plasmids were amplified in Luria–Bertani medium supplemented with 50 μg/ml kanamycin (Bio Basic Inc., Markham, ON, Canada). Plasmid DNA was prepared on columns (NucleoBond PC 500; Macherey-Nagel, Düren, Germany). The identity of the amplified plasmids was confirmed by capillary sequencing (ABI 3730XL; Applied Biosystems) using the sequencing primers 5′-GATCCGCGAGATCCTGAT-3′ (forward) and 5′-CCTATTGGCGTTACTATG-3′ (reverse).

293T cells were plated (1 × 10^4^/well) in 96-well plates. A total of 100 ng of plasmid DNA was cotransfected with miRNA mimic, anti-miRNA or negative controls as described above. Luciferase assays were performed 48 hrs after transfection using the dual-luciferase reporter assay system (Promega). Firefly luciferase activity was normalized to Renilla luciferase expression for each sample. Each experiment was conducted in triplicate.

### Statistical analysis

Statistical analyses were performed with SPSS 19.0 for Windows (SPSS, Chicago, IL, USA). The normality of the data was assessed using the Shapiro–Wilk test. Comparisons between two groups were performed with the two-sample *t*-test or the Mann–Whitney *U*-test for continuous variables and the chi-squared test for categorical variables. For comparisons among >2 groups, one-way anova was performed, and Dunnett's procedure was used for multiple comparisons. Correlation analyses were performed with Pearson's correlation analyses. All of the statistical tests were two-tailed, and *P*-values less than 0.05 were considered statistically significant.

## Results

### The expression of miR-30d and miR-181a is significantly increased in the USLs of POP patients and is inversely correlated with HOXA11 expression

**Fig 1:**
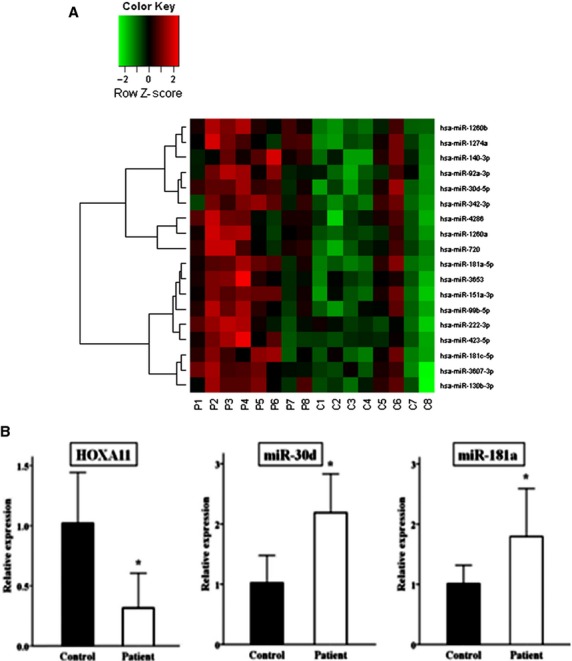
MiRNA expression profiles in the USLs. Microarray revealed that eighteen miRNAs were overexpressed in POP patients compared with controls (*n* = 8/group), as shown in the form of heatmaps (A). qRT-PCR analyses of two miRNAs and HOXA11 expression in the USLs (B). The expression of miR-30d and 181a was significantly increased in women with POP (*n* = 30) compared to controls, whereas HOXA11 mRNA expression was significantly decreased in POP patients *versus* controls (*n* = 30/group). Quantitative data representing the mean ± SD are presented in the bar graph. *P < 0.01 compared with expression in the USLs from the controls.

**Fig 2:**
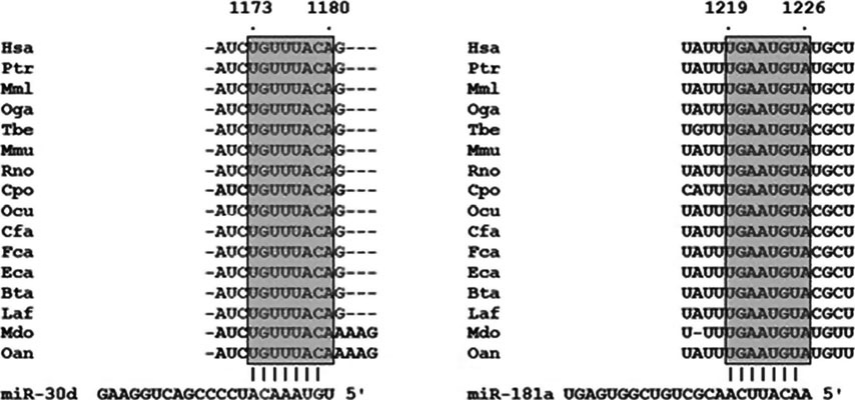
Sequence alignment between miR-30d or 181a and the 3′-UTR of HOXA11 in several species. The seed sequences of HOXA11 3′-UTR targeted by miR-30d and 181a are highly conserved across species (from TargetScan). The number represents the position of the ‘seed region’ matched to miR-30d and 181a within the UTR sequences. *Hsa*, human; *Ptr*, chimpanzee; *Mml*, rhesus; *Oga*, bushbaby; *Tbe*, treeshrew, *Mmu*, mouse; *Rno*, rat; *Cpo*, guinea pig; *Ocu*, rabbit; *Cfa*, dog; *Fca*, cat; *Eca*, horse; *Bta*, cow; *Laf*, elephant; *Mdo*, opossum; *Oan*, platypus.

In an attempt to identify miRNAs involved in HOXA11 regulation, we first compared miRNA expression profiles in the USLs of POP patients and controls using microarray. The miRNAs that were overexpressed differentially in POP patients included miR-222, miR-720, miR-1260a, miR-1260b, miR-99b, miR-3653, miR-92a, miR-423, miR-30d, miR-130b, miR-3607, miR-4286, miR-140, miR-181c, miR-1274a, miR-342, miR-151a and miR-181a (Fig.1A, Table2). Then we searched miRNAs that could regulate HOXA11 using biocomputational prediction algorithms from three different programs (miRanda, TargetScan and PicTar). This approach is known to provide a good balance of sensitivity and specificity and reduce the risk of predicting false positive targets 17. Approximately ten miRNAs were identified as potentially targeting HOXA11 mRNA. The two most notable miRNAs were miR-30d and 181a, which were also significantly overexpressed in the microarray. The alignment of the 3′-UTR of HOXA11 among a wide range of species revealed that the predicted binding sites for miR-30d and 181a are highly conserved during evolution, suggesting the potential importance of these binding sites in HOXA11 (Fig.2).

**Table 2:** Highly overexpressed miRNAs in the USLs of women with POP

miRNA	Patients (mean ± SD)	Controls (mean ± SD)	Fold change	*P*-value
miR-222	8.81 ± 0.73	7.87 ± 0.53	1.91	0.01
miR-720	15.12 ± 0.55	14.21 ± 0.51	1.87	<0.01
miR-1260b	11.91 ± 0.38	11.02 ± 0.38	1.85	<0.01
miR-1260a	12.76 ± 0.57	11.96 ± 0.36	1.75	<0.01
miR-99b	9.44 ± 0.48	8.65 ± 0.55	1.74	<0.01
miR-3653	9.09 ± 0.56	8.31 ± 0.43	1.72	<0.01
miR-92a	10.63 ± 0.46	9.93 ± 0.43	1.62	<0.01
miR-423	8.38 ± 0.59	7.68 ± 0.29	1.61	0.01
miR-30d	10.43 ± 0.30	9.74 ± 0.59	1.61	0.01
miR-130b	7.05 ± 0.37	6.39 ± 0.59	1.58	0.02
miR-3607	6.41 ± 0.32	5.74 ± 0.52	1.58	0.01
miR-4286	13.36 ± 0.41	12.70 ± 0.53	1.58	0.02
miR-140	11.18 ± 0.40	10.55 ± 0.56	1.55	0.02
miR-181c	6.46 ± 0.44	5.83 ± 0.51	1.55	0.02
miR-1274a	11.61 ± 0.39	10.98 ± 0.46	1.55	0.01
miR-342	10.11 ± 0.36	9.49 ± 0.48	1.52	0.01
miR-151a	8.66 ± 0.25	8.07 ± 0.45	1.51	<0.01
miR-181a	9.11 ± 0.27	8.52 ± 0.45	1.50	<0.01

miRNA, microRNA; USL, uterosacral ligament; POP, pelvic organ prolapse.

Next, we performed further qRT-PCR analyses for the remaining samples not used in the microarray analysis to investigate the levels of miR-30d, miR-181a and HOXA11 mRNA expression and the correlation between these miRNAs and HOXA11 expression. Compared to controls, the expression levels of miR-30d and 181a were significantly increased in POP patients, whereas HOXA11 mRNA expression was significantly decreased (Fig.1B). In addition, there were significant inverse correlations between the expression of miR-30d or 181a and HOXA11 mRNA expression (Pearson correlation coefficient = −0.78 and −0.73, respectively; *P* < 0.01). However, we did not find any significant POP-Q stage-related correlation or difference in the expression levels of miR-30d, miR-181 and HOXA11 among POP patients (*P* > 0.05).

### Both miR-30d and miR-181a regulate HOXA11 mRNA and protein levels

**Fig 3:**
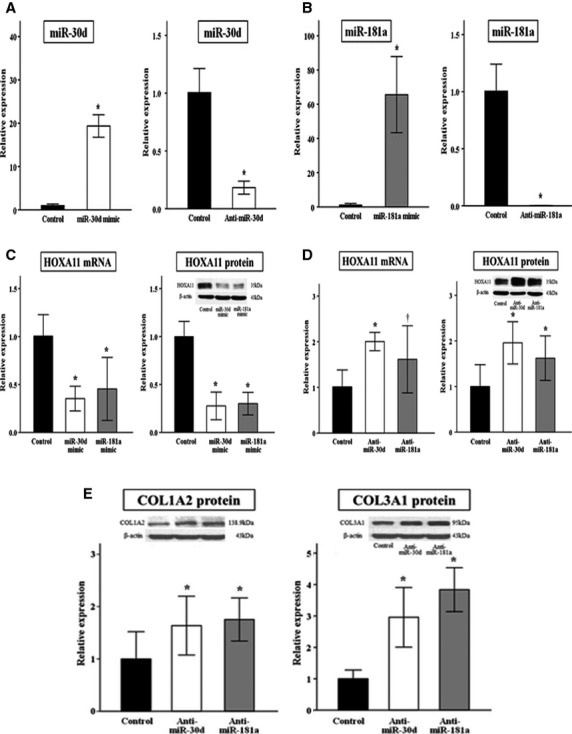
Effects of the overexpression and knockdown of miR-30d or 181a on the expression of HOXA11. qRT-PCR analysis for miR-30d (A) or 181a (B) in 293T cells transfected with a specific miRNA mimic or anti-miRNA, indicating the successful overexpression or knockdown. Transfection with miR-30d or 181a mimic decreased HOXA11 mRNA and protein levels (C). Conversely, transfection with anti-miR-30d or 181a resulted in an increase in HOXA11 levels (D) and collagen production (E). Each experiment was conducted in triplicate. Quantitative data representing the mean ± SD are presented in the bar graph. *P < 0.01, ^†^*P* < 0.05 compared with negative controls. Note that the left and right graph on E have different scales on the *Y*-axis.

We performed a series of functional studies to determine the role of miR-30d and 181a in the regulation of HOXA11. First, whether the overexpression of miR-30d or 181a was sufficient to repress HOXA11 levels was tested. These miRNAs were overexpressed using specific miRNA mimics. qRT-PCR showed that the expression of miR-30d and 181a was significantly increased (Fig.3A and B), and this repressed endogenous HOXA11 mRNA and protein levels in 293T cells (Fig.3C). Next, cultured 293T cells were transfected with anti-miR-30d, anti-miR-181a or a negative control. The successful knockdown of these miRNAs in the 293T cells was confirmed (Fig.3A and B) and demonstrated that the knockdown of miR-30d or 181a enhances HOXA11 mRNA and protein levels, which was accompanied by increased production of collagen type I and III proteins (Fig.3D and E).

### HOXA11 is a direct target of miR-30d and miR-181a

**Fig 4:**
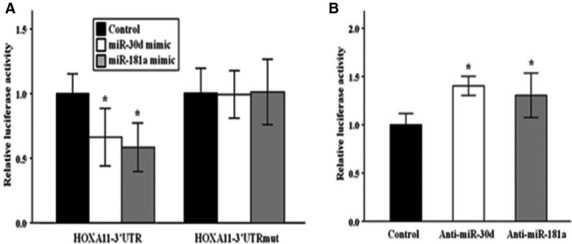
Direct regulation of HOXA11 by miR-30d and 181a. 293T cells were transfected with luciferase reporter plasmid containing 3′-UTR of HOXA11, together with miRNA mimic, anti-miRNA or negative controls. MiR-30d or 181a overexpression significantly decreased the relative luciferase activity of the wild-type 3′-UTR but not the mutant 3′-UTR in which these two miRNA-binding sites were deleted (A). Conversely, knockdown of miR-30d or 181a significantly increased the relative luciferase activity (B). Each experiment was conducted in triplicate. Quantitative data representing the mean ± SD are presented in the bar graph. *P < 0.01 compared with negative controls.

The above findings suggest that HOXA11 is regulated by miR-30d and 181a in the USLs. However, the regulation of HOXA11 by these miRNAs may be indirect. To assess whether miR-30d and 181a can directly alter the expression of HOXA11, a luciferase expression plasmid containing the full-length 3′-UTR of HOXA11 transcript was transfected into 293T cells. Cotransfection of this luciferase expression plasmid with miR-30d or 181a mimic, but not a negative control, resulted in a significant decrease in relative luciferase activity. In addition, the deletion of these two miRNA-binding sites completely abolished miR-30d- and 181a-mediated repression, demonstrating the specificity of repression (Fig.4A).

Because miR-30d and 181a mimics could inhibit HOXA11 3′-UTR luciferase activity, inhibitors to these two miRNAs were assessed to determine if they could exert the opposite effect. Not surprisingly, cotransfection with anti-miR-30d or 181a significantly increased the relative luciferase activity (Fig.4B). Taken together, these results show that both miR-30d and 181a can directly influence HOXA11 through specific binding to its 3′-UTR.

## Discussion

Pelvic floor connective tissues (endopelvic fascia and ligaments) are critical structures that support pelvic organs, along with the levator muscles. They are composed of abundant extracellular matrix, where collagen comprises a large proportion and is responsible for conferring structural integrity and tensile strength 18. Tissue remodelling is a dynamic process, and therefore the balanced turnover of collagen is necessary to maintain the mechanical strength of the tissue. However, previous biochemical studies have demonstrated a decrease in collagen content in the pelvic supportive connective tissues of women with POP compared to women without POP, and this is related to decreased synthesis and increased breakdown by matrix metalloproteinase as well as decreased cellularity, specifically, of fibroblasts 9,19–21. Thus, reversal of this process represents an important therapeutic target in POP management.

Given its vital role in the control of collagen metabolism and cell proliferation 7–9, HOXA11 may serve as a good candidate for the maintenance of collagen homoeostasis in the USLs. HOX genes are predominantly expressed during embryonic development, but also continued expression of HOXA cluster genes is observed in the adult female reproductive tracts 22. However, the expression of HOXA11 mRNA and protein is significantly decreased in the USLs of women with POP 7,9. As a plausible posttranscriptional regulator, we try to identify the specific miRNAs involved in the regulation of HOXA11. Here, we provide several lines of evidence to show that the expression of HOXA11 in USLs is regulated by two miRNAs, miR-30d and miR-181a. First, miR-30d and 181a are overexpressed in women with POP compared to women without POP, and the expression of both miRNAs is inversely correlated with HOXA11 mRNA expression. Second, the overexpression of miR-30d or 181a suppresses HOXA11 mRNA and protein levels in 293T cells, whereas the knockdown of these miRNAs enhances HOXA11 levels and collagen production. Third, these miRNAs can directly influence mRNA levels through specific binding to the 3′-UTR of HOXA11. Taken together, these results indicate that both miR-30d and 181a are important posttranscriptional regulators of HOXA11 and the overexpression of these miRNAs may be a hidden mechanism of the deficient HOXA 11 signalling in the USLs associated with POP.

Because each miRNA can bind and regulate multiple mRNA targets 11, the aberrant expression of these miRNAs may also contribute to the pathogenesis of POP through pathways other than the dysregulation of HOXA11. For example, connective tissue growth factor, a powerful inducer of extracellular matrix synthesis, is known to be a target of miR-30 family 16. Therefore, miR-30d overexpression may limit collagen synthesis by inhibiting the expression of connective tissue growth factor as well as HOXA11. In addition, both miR-30d and 181a are known to play an important role in cellular apoptosis. MiR-30d functions as a proapoptotic inducer 23, and miR-181a directly repress the expression of B cell lymphoma-2 (Bcl-2), an anti-apoptotic protein that interacts with mitochondria 24,25, thereby promoting cellular apoptosis. Notably, increased mitochondrial apoptosis and decreased Bcl-2 expression have been reported in the pelvic supportive tissues of POP patients 26,27. The overexpression of miR-30d and 181a may underlie these changes. The broad effects of miR-30d and 181a in targeting several genes that affect collagen homoeostasis imply that a therapeutic approach to decrease miR-30d and 181a might be beneficial to prevent or restore the decrease in collagen content within the USLs, as revealed by this study.

Although this study suggests key roles for miR-30d and 181a in the control of HOXA11 expression, the overexpression of these miRNAs may not fully explain the decreased HOXA11 expression that is observed in the USLs of women with POP. It is important to note that a direct one-to-one stoichiometric relationship between the levels of miR-30d or 181a and HOXA11 expression does not exist. In the experiments involving the overexpression and knockdown of miR-30d or 181a, the magnitude of the changes in HOXA11 expression was modest compared with the changes in the expression of these miRNAs, suggesting that they are not the sole determinants of HOXA11 mRNA expression. One of possible mechanisms is that HOXA11 can be also controlled by other miRNAs. Indeed, biocomputational prediction algorithms found approximately ten miRNAs potentially targeting HOXA11 mRNA. Instead of assessing all of them, we performed further study only for two miRNAs (miR-30d and 181a) that were overexpressed in the microarray. The coordinate action of several miRNAs may be involved in the HOXA11 deficient signalling in the USLs of POP patients, and this needs to be evaluated in future studies. In addition, these data were derived from *in vitro* cultured 293T cells, which may not reflect complex regulatory interactions *in vivo*. Indeed, pelvic floor fibroblasts express estrogen receptors 28, and the estrogen-estrogen receptor (ER) complex is known to directly bind to regulatory elements of HOXA11 and induce its expression 29. Therefore, dysregulation of miRNAs that target ER may affect HOXA11 expression *in vivo*. It has been shown that ERα protein expression is significantly reduced in both premenopausal and postmenopausal patients with POP 30,31. Among the eighteen miRNAs that were overexpressed in the microarray described in the present study, miR-222 is known to regulate ERα expression at the posttranslational level 32. While this work was in progress, Shi *et al*. also found that miR-222 expression is increased in the USLs of women with POP compared to normal counterparts and that there is an inverse correlation between ERα protein and miR-222 expression 33. In conclusion, based on Shi *et al*.'s study and ours, the data suggest that the cooperative activity of dysregulated miRNAs may contribute to the deficient HOXA11 signalling in the USLs associated with POP. In this regards, the combined treatment of estradiol and anti-miR-222 along with anti-miR-30d or 181a may further increase the expression of HOXA11 protein compared with anti-miR-30d or 181a alone. This needs to be evaluated and confirmed in further study.
